# “When I think about my future, I just see darkness”: How youth exiting homelessness navigate the hazy, liminal space between socioeconomic exclusion and inclusion

**DOI:** 10.17269/s41997-023-00804-2

**Published:** 2023-07-18

**Authors:** Naomi S. Thulien, Stephen W. Hwang, Nicole Kozloff, Rosane Nisenbaum, Alex Akdikmen, Oluwapelumi (Pukky) Fambegbe, Robyn Feraday, Caitlin Mathewson, Matthew Mutamiri, Julia Roglich, Andrea Wang, Micah Zagala, Alexandra Amiri

**Affiliations:** 1 MAP Centre for Urban Health Solutions, Li Ka Shing Knowledge Institute of St Michael’s Hospital, Unity Health Toronto, Toronto, ON Canada; 2 Dalla Lana School of Public Health, University of Toronto, Toronto, ON Canada; 3 Centre for Critical Qualitative Health Research, University of Toronto, Toronto, ON Canada; 4 Division of General Internal Medicine, Department of Medicine, University of Toronto, Toronto, ON Canada; 5 Department of Psychiatry, University of Toronto, Toronto, ON Canada; 6 Slaight Family Centre for Youth in Transition, Centre for Addiction and Mental Health, Toronto, ON Canada; 7 Applied Health Research Centre, Li Ka Shing Knowledge Institute of St Michael’s Hospital, Unity Health Toronto, Toronto, ON Canada; 8 Faculty of Health, York University, Toronto, ON Canada; 9 Mohawk College, Hamilton, ON Canada; 10 Lawrence S. Bloomberg Faculty of Nursing, University of Toronto, Toronto, ON Canada; 11 School of Medicine, Boston University, Boston, MA USA; 12 Temerty Faculty of Medicine, University of Toronto, Toronto, ON Canada

**Keywords:** Youth homelessness, Socioeconomic inclusion, Transition, Critical qualitative methodology, Community-based research, Identity capital, Sans-abrisme des jeunes, inclusion socioéconomique, transition, méthodologie qualitative critique, recherche communautaire, capital identitaire

## Abstract

**Objectives:**

The overarching objective of this mixed methods longitudinal study was to understand whether and how rent subsidies and mentorship influenced socioeconomic inclusion outcomes for youth exiting homelessness. The focus of this paper is on the qualitative objectives, which evolved from a primary focus on exploring how study mentorship was working as a facilitator of socioeconomic inclusion to focusing on how participants navigated the hazy, liminal space between socioeconomic exclusion and inclusion.

**Methods:**

This was a convergent mixed methods study scaffolded by community-based participatory action axiology. The quantitative component is reported elsewhere and involved a 2-year pilot randomized controlled trial where 24 participants received rent subsidies and 13 were randomly assigned a study mentor; proxy indicators of socioeconomic inclusion were measured every 6 months for 2.5 years. Qualitative objectives were explored using a qualitative descriptive design and theoretically framed using critical social theory. The lead author interviewed 12 participants every 6 months for 2.5 years. Qualitative interviews were analyzed using reflexive thematic analysis with an emphasis on critical interpretation.

**Results:**

Navigating the liminal space between socioeconomic exclusion and inclusion was complex and non-linear, and the way youth navigated that journey was more strongly associated with factors like informal mentorship (naturally occurring “coach-like” mentorship) and identity capital (sense of purpose, control, self-efficacy, and self-esteem), rather than whether or not they were assigned a formal study mentor.

**Conclusion:**

A holistic approach integrating coaching and attention to identity capital alongside economic supports may be key to helping youth exiting homelessness achieve socioeconomic inclusion.

## Résumé

**Objectifs:**

L’objectif primordial de cette étude longitudinale à méthodes mixtes était de comprendre si et comment les suppléments au loyer et le mentorat influencent les résultats sur le plan de l’inclusion socioéconomique pour les jeunes qui sortent du sans-abrisme. Notre article porte sur des objectifs qualitatifs; à l’origine, il visait principalement à explorer l’efficacité du mentorat des études comme moyen de faciliter l’inclusion socioéconomique, puis il a évolué en s’attachant à la manière dont les participantes et les participants trouvaient leurs repères dans l’espace liminaire flou entre l’exclusion et l’inclusion socioéconomique.

**Méthode:**

Cette étude à méthodes mixtes convergentes est échafaudée sur l’axiologie de l’action participative communautaire. L’élément quantitatif, qui fait l’objet d’un autre article, a impliqué un essai pilote comparatif randomisé de deux ans où 24 participantes et participants ont reçu des suppléments au loyer, et 13 ont été jumelés de façon aléatoire à un tuteur ou une tutrice scolaire; des indicateurs approximatifs de l’inclusion socioéconomique ont été mesurés tous les six mois pendant deux ans et demi. Les objectifs qualitatifs ont été explorés à l’aide d’un protocole descriptif qualitatif et encadrés théoriquement par la théorie sociale critique. L’autrice principale a interviewé 12 participantes et participants tous les six mois pendant deux ans et demi. Les entretiens qualitatifs ont été analysés en employant l’analyse thématique réflexive et en mettant l’accent sur l’interprétation critique.

**Résultats:**

L’exploration de l’espace liminaire entre l’exclusion et l’inclusion socioéconomique était complexe et non linéaire, et le parcours des jeunes était davantage associé à des facteurs comme le mentorat informel (le mentorat naturel semblable à celui d’un entraîneur ou d’une entraîneuse) et le capital identitaire (le sentiment d’avoir un but, le contrôle, l’auto-efficacité et l’estime de soi) qu’au fait d’avoir ou non été jumelés à un tuteur ou une tutrice dans leurs études.

**Conclusion:**

Une démarche holistique intégrant l’encadrement et l’attention au capital identitaire, en plus des mesures de soutien économique, pourrait être essentielle pour aider les jeunes qui sortent du sans-abrisme à s’intégrer sur le plan socioéconomique.

## Introduction

Research on social and structural inequities that cause and perpetuate youth homelessness in high-income countries typically highlights factors such as familial poverty and dysfunction, mental health challenges, incomplete secondary education, inadequate employment, and discrimination based on race, disability, gender non-conformity, and sexual orientation (Abramovich, 2016; Centrepoint, 2022; Gaetz et al., 2016; Karabanow, 2008; Morton et al., 2017). Consequently, most young people enter and exit homelessness prepared for a life of survival, without adequate tangible and intangible resources needed to thrive (Dej, 2020; Gaetz et al., 2016; Samuels et al., 2019).

The focus of this paper is on social and economic inclusion for young people living in Canada who have experienced homelessness. We utilized a social determinants of health framing to capture both the material and relational nature of social and economic inclusion/exclusion (Popay et al., 2008; Solar & Irwin, 2010). The term “social” refers to factors such as social class, social capital, and social cohesion. The term “economic” refers to factors such as education, occupation, and income. We use the term “socioeconomic” in this paper given the intrinsic and inextricably linked nature of these two concepts. The term “inclusion” denotes equity in health and well-being (the outcome of equitable socioeconomic factors), with “exclusion” denoting the opposite.

There is a small but growing body of literature from longitudinal studies conducted in high-income countries that highlights how underprepared most young people are once they leave homelessness (Kidd et al., 2016; Mayock and Parker, 2019; Thulien et al., 2018). Consequently, even though they are no longer homeless, many still *feel* homeless—an outsider trapped in day-to-day survival without tacit (insider) knowledge as to the steps one needs to take to achieve social and economic inclusion (Brueckner et al., 2011; Kidd et al., 2016; Thulien et al., 2019a). The youth homelessness literature is underdeveloped regarding how many young people exist in this hazy, “in-between space”—the liminal space where one is housed but still feels socioeconomically excluded (Chamberlain & Johnson, 2018)—but emerging data suggest this space is precarious and challenging to navigate, and often results in a return to homelessness.

A stark example of this precarious existence was highlighted in a pan-Canadian survey of 1103 youth accessing services for youth experiencing homelessness, which revealed that 76% reported at least two failed attempts to exit homelessness (Gaetz et al., 2016). Of those, 37% reported at least five attempts (Gaetz et al., 2016). These statistics reflect youth returning to the shelter system for assistance, youth who self-identified as needing help. According to the 2018 Canadian Housing Survey (CHS) of 126,465 households (Statistics Canada, 2019), 18% of Canadians aged 15–29 responsible for making household decisions reported at least one episode of hidden homelessness (e.g., couch surfing) in their lifetime (Uppal, 2022), meaning there are likely thousands of young people who are not seeking help, living in the margins, trapped in a cycle of socioeconomic exclusion.

Understandably, one might identify the provision of stable housing as being the primary solution to assist with transitions out of homelessness; indeed, a common misconception about youth homelessness—sometimes perpetuated by well-intentioned housing advocates—is that housing in and of itself will help youth achieve socioeconomic inclusion. Unfortunately, this is often not the case. For example, in the aforementioned CHS, Uppal (2022) highlights that, even after obtaining housing, individuals who have experienced homelessness—especially visible homelessness (e.g., staying in a homeless shelter)—continue to experience substantially worse socioeconomic outcomes compared to those who have never experienced homelessness. Housing interventions that promote positive youth development and socioeconomic inclusion are urgently needed (Gaetz, 2017; Gaetz et al., 2021); however, a 2020 review of youth (aged 13–25) homelessness interventions conducted in high-income countries concluded, “The field lacks rigorous evaluative evidence of many of the program models on which communities and governments rely to address youth homelessness” (Morton et al., 2020, p. 11).

Prior to initiating the intervention discussed in this paper, some of the authors were involved in a 6-week, 6-session mixed method feasibility study designed to bolster identity capital—purpose, control, self-efficacy, and self-esteem (Côté, 2016)—as a way to foster socioeconomic inclusion for young people (*n* = 19) exiting homelessness (Thulien et al., 2021). Quantitative measures of self-esteem and hope—proxy indicators of socioeconomic inclusion—showed statistically significant improvements 9 months post-intervention compared to baseline. Youth who participated in qualitative focus groups spoke about the importance of a non-pathologizing approach to acquiring new skills—(re)framing their learning as something needed “to have a better life” vs. “to get better” (Thulien et al., 2021, p. 12).

The aforementioned study alongside the lead author’s 10-month critical ethnographic study on socioeconomic inclusion with youth exiting homelessness (Thulien et al., 2018, 2019a) informed the development of the overarching objective of this mixed method longitudinal study, which was to understand whether and how rent subsidies and mentorship influenced socioeconomic inclusion outcomes for youth exiting homelessness. The quantitative objectives were to examine whether proxy indicators of socioeconomic inclusion signaled improvement in the intervention group (rent subsidies + mentorship) compared to the control group (rent subsidies only), and these findings are reported elsewhere (Thulien et al., 2022). The focus of this paper is on the qualitative objectives, which evolved from a primary focus on exploring how study mentorship was working as a facilitator of socioeconomic inclusion to focusing on how participants navigated the liminal space between socioeconomic exclusion and inclusion.

## Methods

The Transitioning Youth Out of Homelessness (TYOH) study was a 2-year, convergent mixed methods, community-based, pilot randomized controlled trial (RCT) with 24 young people from three cities in Ontario, Canada (Thulien et al., 2019b, 2022). Portable rent subsidies (youth could live in a location of their choice; the majority lived in market rent accommodations) ranging from $400.00 CAD to $500.00 CAD per month were provided to all participants for 2 years. Random assignment using a sealed envelope method was used to assign 13 study participants to the intervention (rent subsidies + mentorship). Study mentors were at least 5 years older than study participants and screened and selected by our three community partners.

Mentors were instructed to connect (in person, virtually, or by phone, e-mail, or text message) weekly with their mentees over the 2-year intervention period. Participants engaged in quantitative data collection (*n* = 24) and qualitative data generation (*n* = 12) every 6 months starting at baseline for 2.5 years (until 6 months after the 2-year intervention was over). We used a basic qualitative descriptive design given our primary qualitative objective was to co-construct meaning with participants regarding *how* the intervention was working as a *facilitator* of socioeconomic inclusion (Luciani et al., 2019; Merriam & Tisdell, 2016).

## Reflexivity

The lead author of this paper and principal investigator on this study has over a decade of experience working as a nurse practitioner at Covenant House Toronto—one of the three community partners collaborating on this study. She also drew on her social location as a mixed race (South Asian-European) woman and immigrant with a history of economic precarity, along with her experience conducting two other studies with this population, to help reflect more deeply on the complex and intersecting nature of socioeconomic inclusion.

Several authors on this paper have clinical and volunteer experience working with people experiencing homelessness. Additionally, all of the authors involved in qualitative data generation and analysis were able to identify with the stories of the 12 research participants by virtue of their age and life stage, and/or because of their own experiences of homelessness, addiction, mental health challenges, childhood abuse, and sexual orientation. We believe this “insider-outsider” status allowed us to reach a deeper level of analysis and interpretation, and to theorize the data and offer insights beyond superficial understandings of barriers and facilitators to socioeconomic inclusion (Eakin & Gladstone, 2020).

## Axiology and theoretical framework

The overall study was guided by a commitment to community-based participatory action research (CBPAR) axiology (Israel et al., 2018; Wallerstein et al., 2018):

- Research participants viewed as experts in their own lives.
- Concerted effort to reduce power imbalances between the researchers and the community (e.g., creation of a community advisory board).
- Equal value placed on academic knowledge and experiential knowledge.
- Commitment to co-producing practical, actionable data to build community capacity and improve the lives of the research participants (e.g., creation of a documentary film and animation of study findings: www.searchingforhome.ca).
- Duty to remain invested with the community beyond the life of the research project (see “Discussion” re: TYOH 2.0).

We used critical social theory as our theoretical framework (“analytic glasses”) to guide how we analyzed and interpreted qualitative data. Critical social theory has a social justice orientation with a focus on interrogating how inequitable social structures of power play out in the lives of the underserved (Moosa-Mitha, 2015; Strega, 2015). Utilizing critical social theory as theoretical scaffolding meant we started with individual participant experiences and worked backwards to understand and expose inequitable societal conditions that made it challenging for young people to achieve meaningful socioeconomic inclusion. At the same time, we tried to approach qualitative data co-generation, analysis, and interpretation with humility—speaking *with* rather than *for* participants—to avoid (re)producing oppression, and searched for examples of individual agency and resilience in their stories despite structural inequities (Kirkham & Anderson, 2010).

## Data generation and analysis

From May 2019 to February 2022, the lead author met every 6 months with 12 study participants (four from each city). Seven participants were receiving rent subsidies and mentorship, and five were only receiving rent subsidies. We made the decision to follow a small number of participants over an extended period of time to get a deeper sense of how the mentorship relationships were evolving over time. There were also pragmatic considerations given participants lived in three different cities and the initial intention (prior to pandemic-related restrictions) was to conduct all the interviews in-person. We chose to include participants in both the intervention and the control group because we wanted to compare/contrast their experiences and explore whether there may be factors *other than* study mentorship mediating socioeconomic inclusion.

There were 24 in-person interviews conducted before pandemic-related restrictions required changing to video or phone for the other 47 interviews (one person chose not to participate in their final interview). Interview questions were loosely structured and conversational in nature, focused on socioeconomic inclusion and questions that arose from analysis of prior interviews. Field notes were written immediately after each interview and contained a mixture of what could not be captured through audio recordings (e.g., participant surroundings and a general sense of their well-being) and preliminary analytic insights. All interviews were audio-recorded and then transcribed by a member of the research team.

We used reflexive thematic analysis to help us make sense of the qualitative data (Braun and Clarke, 2022). Briefly, reflexive thematic analysis is *part of* an analytic process comprised of six iterative phases (data familiarization, coding, initial theme generation [patterns anchored by a shared idea/concept], theme development and review, theme refining, and writing up) that *also requires* engagement with theory, reflexivity, and interpretation (Braun and Clarke, 2022). The qualitative research team met monthly for 90–120 min over 2.5 years and was committed to “value-adding” analysis (Eakin & Gladstone, 2020, p.1)—pushing ourselves to go beyond “bare bones”/superficial interpretations (Mykhalovskiy et al., 2018, p. 614) and re-conceptualize/re-frame the issue of socioeconomic inclusion for youth exiting homelessness.

During the team meetings, we discussed what was and was not (Kawabata & Gastaldo, 2015) present in the transcripts (e.g., sometimes participants did not answer how team members might have expected). The lead author would often share emerging analytic insights with study participants, and their feedback was discussed at subsequent team meetings. Analysis and interpretation were primarily inductive (moving from data to conceptualizing—e.g., liminality); however, deductive reasoning (moving from conceptualizing to data) was also employed when we saw familiar theoretical notions (e.g., identity capital) and when we wanted to understand the data through the lens of our emerging conceptual framework. The web-based application Dedoose (Version 9.0.46) was utilized to assist with sorting and coding the qualitative data.

There is a common understanding among qualitative researchers that the main research question one goes into the study with might end up being different than the one that actually gets answered; the question is more like a “compass” than an “anchor” (Eakin & Mykhalovskiy, 2003, p. 190). This was certainly our experience with this study. In the initial set of interviews, we focused on trying to understand the difference between the seven youth receiving rent subsidies and formal mentorship (intervention group) and the five youth only receiving rent subsidies (control group). However, we quickly learned that the journey toward socioeconomic inclusion was complex and non-linear, and the way youth navigated that journey was associated more with factors such as informal mentorship (mentors outside our study) and having strong identity capital—a sense of purpose and belief in their ability to control their destinies (Côté, 2016)—rather than whether or not they were assigned a formal study mentor. In light of these early interpretations of the data, we shifted our orientation to understanding how the young people as a cohort navigated the dim, liminal space of post-homelessness, and the challenges of breaking free from the fog of exclusion.

## Results

The baseline demographics of those participating in qualitative interviews were similar to the overall cohort of study participants (Table 1). The average age of qualitative interview participants was 22 years; most experienced homelessness for the first time before they turned 18. Half self-identified as a woman, and half self-identified as a different race/ethnicity than white. The majority were born in Canada. Just over half had experiences with child welfare involvement. Half had attempted to exit homelessness three or more times; one third of those had tried at least five times. Most had finished high school. A notable number had regular contact with an adult relative, and almost half were able to identify informal mentors (mentors outside the study). Just over half the participants were employed, and most were receiving some form of government income support. All participants reported at least one adverse childhood experience (ACE); a notable majority reported four or more.

**Table 1:** Baseline participant demographics

	Participants, no.	
Characteristic	Total study participants (*n* = 24)	Qualitative interview participants (*n* = 12)
Site		
Toronto	12	6
Hamilton	6	3
St. Catharines	6	3
Age (average years)	21.8	21.8
First time became homeless		
Age (average years)	17.8	17.4
Gender^a^		
Woman	12	6
Man	12	6
Race/ethnicity		
White	10	6
Black	8	4
Asian	2	0
Indigenous	2	1
Different choice	2	1
Born in Canada	20	11
Child welfare involvement	13	7
Attempts to live on own after being homeless		
1–2	13	6
3+	11	6
Number of attempts to exit homelessness		
1–2	13	6
3–4	4	2
5+	7	4
Highest education level		
Less than high school	8	5
Completed high school	8	4
Some or completed post-secondary	8	3
Regular contact with adult relative	19	8
Informal adult mentor^b^	9	5
Employed	14	7
Social assistance^c^	18	8
ACE^d^		
1–3	6	2
4–9	17	10

^a^All participants were given the option of choosing “different gender identity”; no one chose this option

^b^Not a relative and someone outside the social service sector (i.e., not a case worker)

^c^Government welfare payments to cover basic needs and housing costs, ranging from $730.00 CAD/month to $1170.00 CAD/month. It was possible for participants to be both employed and on social assistance; however, employment income above $200.00 CAD/month resulted in a reduction of social assistance payments by $0.50 for every $1.00 of employment income

^d^ACE: adverse childhood experiences questionnaire (ACEs Aware, 2020) completed at 24 months during quantitative data collection; *n* = 23 (one participant lost to follow-up at 24 months)

**Fig. 1:**
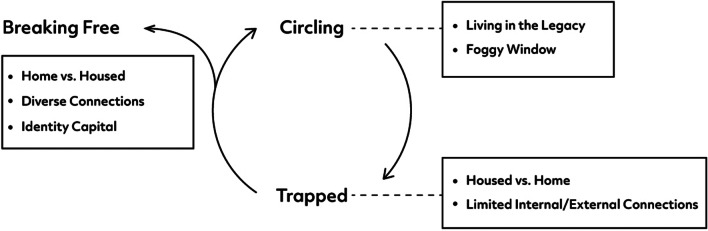
Navigating the liminal space between socioeconomic exclusion and inclusion

We generated three central themes from our qualitative analysis and interpretation: circling, trapped, and breaking free (Fig. 1). Pseudonyms chosen by participants are used throughout. Lengthy pauses are indicated by … and […] is used to denote missing text.

### Circling: living in the legacy

“*When I think about my future, I just see darkness. I don’t know what to expect from it because I’m not really planning for it*.” (Mazi, baseline interview)

At various points during the study—especially at the beginning—we noticed many participants either had a hard time articulating their goals or described their future using words like “dark”, “fog”, “hazy”, “fuzzy”, “circle-y”, and “murky”—like they were trying to look through a foggy window. Many expressed a sense of purposelessness and mundaneness to their existence and a feeling that the future was either too overwhelming to consider or simply out of their control. The majority of participants grew up in homes with varying degrees of family dysfunction and financial stress, and many described traumatizing primary and secondary school experiences such as bullying and negative messages about their cognitive ability. Most continued to live in the legacy of these childhood experiences—thrust into adult roles as teenagers (some even earlier), consumed with making ends meet rather than planning for the future:

I think maybe when you’re in a position where you have to take care of yourself, and that causes you to be paycheck to paycheck, and you’re struggling even though you don’t have other responsibilities, you just kind of feel like you’re not going to have a chance to actually pursue what you actually may want, or actually want in life, because you have to worry about these little things, these minor things. Already, it’s like you don’t have the fair chance to actually look towards an actual bright future for yourself. (Monique, 6-month interview)

At the time of this interview, Monique had consistent full-time (minimum wage) employment and was housed; on the outside, she had “successfully” exited homelessness. Despite these achievements, she demonstrated an acute awareness of forced self-reliance and inequitable life chances. The experience of navigating a post-homelessness existence was also confounded by structures that seemed almost intentionally cruel and challenging to maneuver:

I went into this circle of being told to obtain stable housing to be able to get the children back to my care, but [social welfare program] is not giving me enough to do that, so I went in this circle of not being able to get housing, and then [child protective services] won’t return the children until I can get housing, so it was a constant circle I was going through. […] So now [that she is receiving rent subsidies through the study] I can show them that I am doing consistent groceries and that I have a bus pass to be able to get to my appointments. I’m kinda like showing stability and the fact that I am able to do what I need to do. Then when they have my visit showing the adequacy, it shows that I have prepared for the children for when they come. (Alice, baseline interview)

Notable from this excerpt is that, despite living in poverty and navigating life on her own, Alice was expected to present the illusion of stability and demonstrate “adequacy” to those who have the power to take away her children for good. Almost 2.5 years later, Alice was still living in a two-bedroom apartment (required by child protective services to prove she had room for the children) trying to get her children back and demonstrate adequacy:

How am I supposed to pay rent for a two-bedroom place on a single person income? My single person income is $1,100 and my rent is $960 and change. […] I’m constantly stuck in the catch-22. (Alice, 30-month interview)

### Trapped: searching for connection and home

“*Home is not a place – it’s not just a place where you sleep. It’s a place where you feel comfortable to be who you want to be. It’s not acting for someone else. It’s not worrying about who’s listening or what you have to say or how you have to dress or how you have to act. It’s just some place that you can exist as you*.” (Colin, 12-month interview)

As we journeyed with the young people over the course of 2.5 years, we saw that some seemed to be stuck circling; even though they were housed, they did not feel at home. As we dug deeper into the experience of housed vs. home, we came to understand that “housed” was a tangible physical structure, but “home” was an intangible sense of physical/mental safety and connection to oneself and to others—an awareness of belonging, of inclusion. In other words, house and home were related but not synonymous concepts.

In this excerpt from Colin, he was describing his experience of searching for home while existing in transitional, in-between spaces—couch surfing with relatives and friends after high school and then moving into the first space of his own, where he did not feel safe or connected. By the end of the study, home for Colin meant reconciling with his father and then renting a three-bedroom townhome together. For other study participants, even after 2.5 years of relative housing stability, they were still searching for connection and home:

There’s like some weird sense of home there [homeless shelter] that neither of us have had anywhere else. I have people who feel like home […]. Home is not really a place. (Riccio, 30-month interview)

It is remarkable that, despite the fact that Riccio had been living away from the shelter for over 3 years, it was still the only physical space that felt like home. In this excerpt, she was including the experience of her partner, who was living in a shelter at the time. Similar to Monique who displayed external markers of success (steady employment and housing), Riccio was pursuing post-secondary education, volunteering as a peer mentor in the homelessness sector, and living in market rent housing. Still, on the inside, part of her longed for the only physical place that ever felt like home: the homeless shelter. Like Colin, Riccio explained that a sense of home had more to do with connection than a physical structure.

In addition to the uneasy sense of not being settled, young people trapped in the dark fog of exclusion generally had limited insight into how to move forward and extremely limited social connections. Jordan described the complicated entanglement between self-understanding and socioeconomic inequities he was still navigating 2 years into the study while living with his parents, partner, and new baby:

[Having a baby] makes me feel like I have more of a purpose now, ‘cause like beforehand, I didn’t really know what my point was in life, to be honest. […] We get about six or seven hundred dollars in child tax credit now since I sent in the paperwork […] It couldn’t have come at a better time because we were almost out of baby food and formula and stuff. My parents need us to [help financially] – like last month for instance, my dad made too much at work, so my mom got less from ODSP [Ontario Disability Support Program; enhanced financial resources for those with a diagnosed disability]. So, they needed the full $600 [for rent] in one shot rather than $300 in the beginning and $300 in the middle […] I applied for ODSP, but they declined me twice, three times. I guess my anxiety and stuff wasn’t good enough. (Jordan, 24-month interview)

Like Colin, Jordan moved in with his parents during the study and was helping pay the rent. It is clear from this excerpt that money was tight; this was the only existence Jordan had ever known. He had not finished high school and felt overwhelmed at the thought of applying for and holding down a job. Jordan was trapped in a generational cycle of socioeconomic exclusion. To help relieve some of the financial strain, he sought the same government supports that had been provided to his mother. His comment that his anxiety “wasn’t good enough” is similar to Alice’s comment about “adequacy” and highlights the burden of socioeconomic inclusion carried by many participants as they navigated a post-homelessness existence. Six months later, Jordan was still unclear how to move forward when asked to consider where he might be in 5 years:

All I can think of for an answer to that one is not here. It needs to be different […] but I can’t quite clearly see what it’s going to look like […] My family operates on something we call chaos theory. I thrive on the quote unquote chaos of not really having a plan. […] for the most part, if I plan something out, nothing works to my plan. (Jordan, 30-month interview)

Jordan’s comments from two different points during the study highlight three intrinsically connected ideas we heard from others who seemed trapped: (1) a sense of not being settled, not being home; (2) limited understanding as to why they existed; how they might connect with and contribute to society; and (3) even if they did gain an awareness of purpose (like Jordan had as a new father), socioeconomic inequities and an unclear vision made it challenging to have a sense of power and control over that purpose. Jordan’s use of the word “chaos” (i.e., confusion) and belief that his plans do not generally come to fruition are particularly insightful and help illuminate the mindset of participants trapped in a cycle of socioeconomic exclusion.

### Breaking free: the power of connection and identity

“*I think spirituality is really important because it’s an understanding of ourselves. I feel like when we don’t understand what we’re doing here and who we are it makes it really difficult to navigate because you don’t even understand what you’re navigating for in the first place – especially when you’re not really pleased with what’s going on and then you feel kind of like you’re out of control and you’re trying to grasp control, but you don’t really believe that you have control, and it’s just not a great place to be in when you’re actually so much more powerful than you know*.” (Monique, 30 months)

Over the course of 2.5 years, we saw some of the young people begin to break free from the fog of exclusion. While this was not always a linear path, we observed a pattern of connection—spiritual, internal, and external—among those on the journey to inclusion. That sense of connection was intrinsically linked to an awareness of being “home” and possessing strong identity capital—a sense of purpose, control, self-efficacy, and self-esteem (Côté, 2016). For Monique, connecting to a higher power, self-understanding, and a sense of purpose were key to healing feelings of powerlessness (note the number of times she uses the word “control”) that she described (above) in her 6-month interview. Others shared similar illuminating insights about the power of connection:

I’ve been working a lot. I’ve been getting to know a lot of my coworkers, so it’s been alright. It’s a really small town out here so everybody knows each other. [My job is] unionized so people that work there for like 10 to 15 years, they know each other. They are all good people; I like working with them. […] I use work like it’s an antidepressant […] It makes me feel productive, like I’m actually contributing to society. […] I got a lot of relatives that have been on disability their whole lives. Like, it’s not a good way to live your life, right? Like nobody’s ever going to be happy like that. Really, it’s an endless cycle. (Robin, 30-month interview)

In this excerpt, Robin articulates the inextricable link between connection, mental well-being, and a sense of purpose. Robin began experiencing homelessness as a teenager—living under a bridge at one point—battled addiction and struggled with severe post-traumatic stress disorder. At his baseline interview, he described his one-room accommodation as a “prison cell” where “mice watch me sleep” and described himself as “a worthless piece of shit” with a general dislike/distrust of others. In baseline interview field notes, the lead author described his appearance as “disheveled” and noted his clothing, hair, and hands appeared unwashed. By his 12-month interview, Robin had moved in with his mom who was living in a small community, and by his 24-month interview, he had found full-time employment in a factory that offered a stable job and a living wage salary. At his 30-month interview, he agreed to a video call (the 12-, 18-, and 24-month interviews were done over the phone), and the lead author noted in her field notes how different he looked compared to when they first met—he was well groomed and seemed happy. It was inspiring to witness Robin’s journey, which would likely have gone a different direction if not for the connection with family, community, and employment.

Diverse connections—spiritual, internal, and external—helped foster a sense of purpose and healthy self-esteem, and in return, having a sense of purpose and healthy self-esteem fostered diverse connections:

Sometimes when I’m feeling down or when something happens, I call people around me. I have people like [friend] and I call her sometimes. And I call some of the church members. My pastor, she has my number. She calls me all the time. I call her any time. So, I call her [the pastor], I tell her what happened. And she’ll be like, ‘Oh don’t worry, it will be fine. Everybody goes through this.’ Blah blah blah. ‘We’ll pray for you.’ I’ll be like, ‘Okay thank you so much.’ And then my mind will kind of lift up again. And some students I met in school in first semester, they were already in their second or third semester, so I talked to them. They’ll be like, ‘This is how it is. Just don’t give up. You should be fine. You’ll be okay.’ You need positive people around you. Like even [former outreach worker from the shelter] too, I talk to her, and she’ll be like, ‘Sara don’t worry [laughs], you’ll get through it. Yeah, I always call people when I’m having a bad time. I call [former outreach worker], I call my friends, and people in the church. I’ll be like, ‘Oh Jesus, how can I do this?’ They’ll be like, ‘Don’t worry; you will do it.’ (Sara, 24-month interview)

Sara had come to Canada alone as a teenage refugee and spent several years working for minimum wage and living precariously while upgrading her high school education. When she enrolled in the study, she was embarking on a 2-year practical nursing diploma, which she finished, graduating on the Dean’s list. While Sara definitely had a sense of purpose, life was challenging at times, and she was sometimes tempted to give up. Instead, she reached out to her strong network of informal mentors—friends, church members, fellow students, and her former outreach worker—for encouragement and support. Two insights are notable from this excerpt: (1) an understanding of the need to be surrounded by positive people and (2) most of the time, the impetus was on Sara to reach out. In other words, there was an intentionality—a strategy—to Sara’s journey and a symbiotic relationship between identity, connection, and the ability to break through the liminality of post-homelessness and reach toward a sense of home.

## Discussion

This 2.5-year study offers a unique longitudinal perspective on the tremendous inner strength and momentum needed to navigate through and break free from the hazy, liminal space between socioeconomic exclusion and inclusion. Crucially, these experiences existed despite the provision of portable rent subsidies for 2 years, and regardless of being assigned a formal study mentor. These qualitative insights align with our quantitative data that showed no statistically significant improvements in proxy indicators of socioeconomic inclusion in the intervention group (rent subsidies + mentorship) relative to the control group (rent subsidies only) 18 months post-randomization (Thulien et al., 2022).

Socioeconomic inequities along with negative explicit and implicit messages about who these young people were and what they might become set them up to remain in their liminal post-homelessness existence—to succumb to the path of least resistance—rather than forging ahead. Those able to break free from the fog had a sense of purposefulness to their existence, a feeling of being home—belonging to themselves—not wholly dependent on physical accommodation, and were surrounded by diverse connections that helped guide and encourage them on their journey. We observed the potential positive impact of diverse connections in our quantitative data as well; ancillary analysis suggested those with informal mentors at baseline—found in places like school, work, and faith communities—felt significantly more psychologically integrated into the community at 18 months relative to those with no informal mentors at baseline (Thulien et al., 2022). Indeed, peer-reviewed evidence on informal (or “natural”) mentors signals the favourable influence of these “coach-like” connections on underserved youth such as those with current or past experiences with foster care or homelessness (Dang & Miller, 2013; Thompson et al., 2016; Van Dam et al., 2018).

The importance of shifting focus to fostering a sense of home (an intangible feeling of connection and belonging) vs. focusing primarily on housing (a tangible physical structure) is a crucial learning from this study and challenges individual-oriented conceptualizations of what young people exiting homelessness require for meaningful socioeconomic inclusion. An overemphasis on the latter may explain why systematic reviews of qualitative and quantitative studies and rigorous trials of housing-focused interventions (e.g., Housing First) conducted in high-income countries show that it is challenging to demonstrate significant improvements in outcomes beyond housing stability compared with treatment as usual (Aubry et al., 2020; Chen et al., 2022; Kozloff et al., 2016; Marshall et al., 2022; Stergiopolous et al., 2019). Taken together, these findings indicate the urgent need to focus on outcomes beyond housing stability, especially for young people attempting to transition out of homelessness.

The importance of utilizing identity capital to help navigate through and break free from the post-homelessness fog was a fundamental takeaway from this longitudinal study and has important implications for front-line practice and future interventions. There is a socioeconomic “grooming” that begins at birth (Browman et al., 2019a; Oyserman & Destin, 2010), and most participants in our study—74% of whom had an ACE score of at least four, meaning they were essentially predisposed to poor health outcomes, including homelessness (Destin, 2019; Liu et al., 2021)—were groomed for exclusion. When viewed from this perspective, it becomes evident that individual-level interventions addressing inequitable *internal* resources such as identity capital are just as—if not more—important as addressing inequitable external resources such as affordable housing for youth exiting homelessness.

Intervening on some or all components of identity capital to mediate socioeconomic inclusion for youth who have experienced homelessness is a nascent area of research, but there are some promising transferrable findings from interventions with youth who were currently experiencing homelessness and interventions where youth may not have experienced homelessness but were from low socioeconomic backgrounds (Browman et al., 2019b; Cumming et al., 2022; Destin et al., 2021; Krabbenborg et al., 2017; Slesnick et al., 2016). We plan to build on this learning and conduct a 1-year RCT with youth exiting homelessness, offering rent subsidies to all and randomizing half to a coach (mimicking informal mentorship and targeting identity capital) and co-designed (with youth from this study) leadership program. In keeping with CBPAR principles, we are collaborating with the same community partners and working with young people from this study to co-design the upcoming study—TYOH 2.0. Additionally, three young people from this study are paid participants on the TYOH 2.0 advisory board.

### Limitations

More than half of the interviews took place over the phone or virtually because of the COVID-19 pandemic. In addition to being cognisant that these data were generated at a unique time in history (i.e., participants’ responses may be different post-pandemic), not being able to meet in-person limited the ability of the lead author to fully engage with participants and utilize non-verbal cues as data in the analysis and interpretation. Also, all of the participants lived in urban areas, the majority were born in Canada, and more than half had completed high school. Youth living in rural/remote regions, born outside of Canada, and having less formal education may experience exiting homelessness differently.

## Conclusion

Despite receiving rent subsidies and achieving relative housing stability, participants’ post-homelessness existence was marked by exclusion, and many were not equipped with the tools to navigate this dim, liminal space. Utilizing a holistic understanding of home (facilitating connection and belonging along with housing) and fostering identity capital may be key to helping young people achieve socioeconomic inclusion.

## Contributions to knowledge

What does this study add to existing knowledge?

- A unique longitudinal perspective on youth post-homelessness existence.
- Conceptual insights regarding the challenges of exiting homelessness and navigating the liminal space between socioeconomic exclusion and inclusion.
- An enhanced understanding of the internal and external resources required to foster socioeconomic inclusion.

What are the key implications for public health interventions, practice, or policy?

- Rigorous interventions with a primary outcome of socioeconomic inclusion (not housing stability) underpinned by a holistic understanding of home for youth exiting homelessness are urgently needed.
- Youth exiting homelessness require a feeling of home (not just a house), diverse connections (especially informal/natural connections), and a sense of purpose and control (identity capital); failure to address all three of these fundamental areas will likely leave youth trapped in a cycle of socioeconomic exclusion.

## References

[CR1] Abramovich A (2016). Preventing, reducing and ending LGBTQ2S youth homelessness: The need for targeted strategies. Social Inclusion.

[CR2] ACEs Aware. (2020). *Adverse childhood experience questionnaire for adults.* Acesaware.org. https://www.acesaware.org/wp-content/uploads/2020/02/ACE-Questionnaire-for-Adults-Identified-English.pdf

[CR3] Aubry T, Bloch G, Brcic V, Saad A, Magwood O, Abdalla T, Alkhateeb Q, Xie E, Mathew C, Hannigan T, Costello C, Thavorn K, Stergiopoulos V, Tugwell P, Pottie K (2020). Effectiveness of permanent supportive housing and income assistance interventions for homeless individuals in high-income countries: A systematic review. The Lancet. Public Health.

[CR4] Braun V, Clarke V (2022). * Thematic analysis: A practical guide *.

[CR5] Browman AS, Destin M, Kearney MS, Levine PB (2019). How economic inequality shapes mobility expectations and behaviour in disadvantaged youth. Perspective.

[CR6] Browman AS, Svoboda RC, Destin M (2019). A belief in socioeconomic mobility promotes the development of academically motivating identities among low-socioeconomic status youth. Self and Identity.

[CR7] Brueckner M, Green M, Saggers S (2011). The trappings of home: Young homeless people’s transitions towards independent living. Housing Studies.

[CR8] Centrepoint. (2022). *Policy and research: Our research*. https://centrepoint.org.uk/what-we-do/policy-and-research/our-research/

[CR9] Chamberlain C, Johnson G (2018). From long-term homelessness to stable housing: Investigating ‘liminality’. Housing Studies.

[CR10] Chen, K. L., Miake-Lye, I. M., Begashaw, M. M., Zimmerman, F. J., Larkin, J., McGrath, E. L., & Shekelle, P. G. (2022). Association of promoting housing affordability and stability with improved health outcomes: A systematic review. *JAMA Network Open, 5*(11), 1–15. 10.1001/jamanetworkopen.2022.39860 10.1001/jamanetworkopen.2022.39860 PMC9631101 36322083

[CR11] Côté, J. (2016). The identity capital model: A handbook of theory, methods, and findings. *Sociology Publications, 38*. 10.13140/RG.2.1.4202.9046

[CR12] Cumming J, Clarke FJ, Holland MJG, Parry BJ, Quinton ML, Cooley SJ (2022). A feasibility study of the My Strengths Training for Life™ (MST4Life™) program for young people experiencing homelessness. International Journal of Environmental Research and Public Health.

[CR13] Dang MT, Miller E (2013). Characteristics of natural mentoring relationships from the perspectives of homeless youth. Journal of Child and Adolescent Psychiatric Nursing.

[CR14] Dej E (2020). *A complex exile: Homelessness and social exclusion in Canada*.

[CR15] Destin M (2019). Socioeconomic mobility, identity, and health: Experiences that influence immunology and implications for intervention. American Psychologist.

[CR16] Destin M, Debrosse R, Silverman DM (2021). An experimental demonstration of the positive consequences of guiding students to conceptualize education as connection. Journal of Adolescence.

[CR17] Eakin JM, Gladstone B (2020). “Value-adding” analysis: Doing more with qualitative data. International Journal of Qualitative Methods.

[CR18] Eakin JM, Mykhalovskiy E (2003). Reframing the evaluation of qualitative health research: Reflections on a review of appraisal guidelines in the health sciences. Journal of Evaluation in Clinical Practice.

[CR19] Gaetz, S. (2017). *THIS is housing first for youth: A program model guide*. Toronto: Canadian Observatory on Homelessness Press. https://www.homelesshub.ca/sites/default/files/COH-AWH-HF4Y.pdf

[CR20] Gaetz, S., O’Grady, B., Kidd, S., & Schwan, K. (2016). *Without a home: The national youth homelessness survey*. Toronto: Canadian Observatory on Homelessness Press. https://homelesshub.ca/sites/default/files/WithoutAHome-final.pdf

[CR21] Gaetz, S., Water, H., & Story, C. (2021). *THIS is housing first for youth. Part 1 – Program model guide*. Toronto, ON: Canadian Observatory on Homelessness Press. https://www.homelesshub.ca/sites/default/files/HF4Y-Program-Guide-Jul-15.pdf

[CR22] Israel, B. A., Schulz, A. J., Parker, E. A., Becker, A. B., Allen, A. J., Guzman, J. R., Lichtenstein, R. (2018). Critical issues in developing and following CBPR principles. In, N. Wallerstein, B. Duran, J. G. Oetzel, and M. Minkler. (Eds.) *Community-based participatory research for health: Advancing social and health equity*, 3^rd^ ed., (pp. 2094–2102). Jossey-Bass.

[CR23] Kawabata, M., & Gastaldo, D. (2015). The less said, the better: Interpreting silence in qualitative research. *International Journal of Qualitative Methods, 14*(4). 10.1177/1609406915618123

[CR24] Karabanow J (2008). *Being young and homeless: Understanding how youth enter and exit street life*.

[CR25] Kidd SA, Frederick T, Karabanow J, Hughes J, Naylor T, Barbic S (2016). A mixed methods study of recently homeless youth efforts to sustain housing and stability. Child and Adolescent Social Work Journal.

[CR26] Kirkham SR, Anderson JM (2010). The advocate-analyst dialectic in critical and postcolonial feminist research: Reconciling tensions around scientific integrity. Advances in Nursing Science.

[CR27] Kozloff N, Adair CE, Palma Lazgare LI, Poremski D, Cheung AH, Sandu R, Stergiopoulos V (2016). “Housing First” for homeless youth with mental illness. Pediatrics.

[CR28] Krabbenborg MAM, Boersma SN, van de Veld WM, Vollebergh WAM, Wolf JRLM (2017). A cluster randomized controlled trial testing the effectiveness of houvast: A strengths-based intervention for homeless young adults. Research on Social Work Practice.

[CR29] Liu M, Luong L, Lachaud J, Edalati H, Reeves A, Hwang SW (2021). Adverse childhood experiences and related outcomes among adults experiencing homelessness: A systematic review and meta-analysis. The Lancet.

[CR30] Luciani M, Campbell K, Tschirhart H, Ausili D, Jack SM (2019). How to design a qualitative health research study. Part 1: Design and purposeful sampling considerations. Professioni Infermieristiche.

[CR31] Marshall, C. A., Easton, C., Phillips, B., Boland, L., Isard, R., Holmes, J., Shanoff, C., Hawksley, K., Landry, T., Goldszmidt, R., Aryobi, S., Plett, P., & Oudshoorn, A. (2022). Experiences of transitioning from homelessness: a systematic review and meta-aggregation of qualitative studies conducted in middle to high income countries. *Journal of Social Distress and Homelessness, *1–22. 10.1080/10530789.2022.2141868

[CR32] Mayock P, Parker S (2019). Homeless young people ‘strategizing’ a route to housing stability: Service fatigue, exiting attempts and living ‘off grid’. Housing Studies.

[CR33] Merriam, S.B., & Tisdell, E.J. (2016). Chapter 2: Six common qualitative research designs. *Qualitative research: A guide to design and implementation* (4th ed., pp. 22-42). Jossey-Bass.

[CR34] Moosa-Mitha M, Brown L, Strega S (2015). Situating anti-oppressive theories within critical and difference-centered perspectives. * Research as resistance: Revisiting critical, Indigenous, & anti-oppressive approaches *.

[CR35] Morton M, Dworsky A, Matjasko JL, Curry SR, Schlueter D, Chávez R, Farrell AF (2017). Prevalence and correlates of youth homelessness in the United States. Journal of Adolescent Health.

[CR36] Morton MH, Kugley S, Epstein R, Farrell A (2020). Interventions for youth homelessness: A systematic review of effectiveness studies. Children and Youth Services Review.

[CR37] Mykhalovskiy E, Eakin J, Beagan B, Beausoleil N, Gibson BE, Macdonald ME, Rock MJ (2018). Beyond bare bones: Critical, theoretically engaged qualitative research in public health. Canadian Journal of Public Health. Revue Canadienne de Sante Publique.

[CR38] Oyserman D, Destin M (2010). Identity-based motivation: Implications for intervention. The Counselling Psychologist.

[CR39] Popay, J., Escorel, S., Hernandez, M., Johnston, H., Mathieson, J., & Rispel, L. (2008). *Understanding and tackling social exclusion: Final report to the WHO Commission on Social Determinants of Health from the Social Exclusion Knowledge Network*. https://cdn.who.int/media/docs/default-source/documents/social-determinants-of-health/social-exclusion-knowledge-network-2007.pdf?sfvrsn=6b13662_3

[CR40] Samuels, G. M., Cerven, C., Curry, S., Robinson, S. R., & Patel, S. (2019). *Missed opportunities in youth pathways through homelessness*. Chapin Hall at the University of Chicago. https://www.chapinhall.org/wp-content/uploads/ChapinHall_VoYC_Youth-Pathways-FINAL.pdf

[CR41] Slesnick N, Zhang J, Brakenhoff B (2016). Personal control and service connection as paths to improved mental health and exiting homelessness among severely marginalized homeless youth. Children and Youth Services Review.

[CR42] Solar, O., & Irwin, A. (2010). *A conceptual framework for action on the social determinants of health: Social determinants of health discussion paper 2.* Geneva, Switzerland: World Health Organization Press. https://apps.who.int/iris/bitstream/handle/10665/44489/9789241500852_eng.pdf?sequence=1&isAllowed=y

[CR43] Statistics Canada. (2019). *Canadian housing survey*. https://www23.statcan.gc.ca/imdb/p2SV.pl?Function=getSurvey&Id=793713#a3

[CR44] Stergiopoulos V, Meja-Lancheros C, Nisenbaum R, Wang, R., Lachaud, J., O’Campo, P., Hwang, S. W. (2019). Long-term effects of rent supplements and mental health support services on housing and health outcomes of homeless adults with mental illness: Extension study of the At Home/Chez Soi randomised controlled trial. The Lancet.

[CR45] Strega S, Brown L, Strega S (2015). The view from the poststructural margins: Epistemology and methodology reconsidered. * Research as resistance: Revisiting critical, Indigenous, & anti-oppressive approaches *.

[CR46] Thompson AE, Greeson JKP, Brunsink AM (2016). Natural mentoring among older youth in and aging out of foster care: A systematic review. Children and Youth Services Review.

[CR47] Thulien NS, Gastaldo D, Hwang SW, McCay E (2018). The elusive goal of social integration: A critical examination of the socio-economic and psychosocial consequences experienced by homeless young people who obtain housing. Canadian Journal of Public Health.

[CR48] Thulien NS, Gastaldo D, McCay E, Hwang SW (2019). “I want to be able to show everyone that it is possible to go from being nothing in the world to being something”: Identity as a determinant of social integration. Children and Youth Services Review.

[CR49] Thulien NS, Kozloff N, McCay E, Nisenbaum R, Wang A, Hwang SW (2019). Evaluating the effects of a rent subsidy and mentoring intervention for youth transitioning out of homelessness: Protocol for a mixed methods, community-based pilot randomized controlled trial. JMIR Research Protocols.

[CR50] Thulien NS, Wang A, Mathewson C, Wang R, Hwang SW (2021). Tackling exclusion: A pilot mixed method quasi-experimental identity capital intervention for young people exiting homelessness. PLoS One.

[CR51] Thulien NS, Amiri A, Hwang SW, Kozloff N, Wang A, Akdikmen A, Roglich J, Nisenbaum R (2022). Effect of portable rent subsidies and mentorship on socioeconomic inclusion for young people exiting homelessness: A community-based pilot randomized clinical trial. JAMA Network Open.

[CR52] Uppal, S. (2022). *A portrait of Canadians who have been homeless*. Statistics Canada. https://www150.statcan.gc.ca/n1/en/pub/75-006-x/2022001/article/00002-eng.pdf?st=R3KsvbcB

[CR53] Van Dam L, Smit D, Wildschut SJT, Rhodes M, Assink M, Stams GJJM (2018). Does natural mentoring matter? A multilevel meta-analysis on the association between natural mentoring and youth outcomes. American Journal of Community Psychology.

[CR54] Wallerstein, N., Duran, B., Oetzel, J. G., & Minkler, M. (2018). *Community-based participatory research for health: Advancing social and health equity* (3rd ed.). Jossey-Bass.

